# Red fluorescent proteins for imaging *Zymoseptoria tritici* during invasion of wheat^☆^

**DOI:** 10.1016/j.fgb.2015.03.025

**Published:** 2015-06

**Authors:** M. Schuster, S. Kilaru, M. Guo, M. Sommerauer, C. Lin, G. Steinberg

**Affiliations:** a Biosciences, University of Exeter, Exeter EX4 4QD, UK; b AHF Analysentechnik AG, Kohlplattenweg 18, DE-72074 Tübingen, Germany; c Mathematics, University of Exeter, Exeter EX4 3QF, UK

**Keywords:** FPs, fluorescent proteins, RFP, red fluorescent protein, mCherry, monomeric cherry, TagRFP, monomeric red (orange) fluorescent protein, tub2, α tubulin, mRFP, monomeric red fluorescent protein, eGFP, enhanced green fluorescent protein, tdTomato, tandem dimeric red fluorescent protein, sdi1, succinate dehydrogenase 1, dpi, days post infection, ROI, region of interest, *n*, sample size, Red fluorescent protein, Colocalization, Protein localization, Wheat pathogenic fungus, *Septoria tritici* blotch, *Mycosphaerella graminicola*

## Abstract

The use of fluorescent proteins (FPs) in plant pathogenic fungi provides valuable insight into their intracellular dynamics, cell organization and invasion mechanisms. Compared with green-fluorescent proteins, their red-fluorescent “cousins” show generally lower fluorescent signal intensity and increased photo-bleaching. However, the combined usage of red and green fluorescent proteins allows powerful insight in co-localization studies. Efficient signal detection requires a bright red-fluorescent protein (RFP), combined with a suitable corresponding filter set. We provide a set of four vectors, suitable for yeast recombination-based cloning that carries mRFP, TagRFP, mCherry and tdTomato. These vectors confer carboxin resistance after targeted single-copy integration into the *sdi1* locus of *Zymoseptoria tritici*. Expression of the RFPs does not affect virulence of this wheat pathogen. We tested all four RFPs in combination with four epi-fluorescence filter sets and in confocal laser scanning microscopy, both *in* and *ex planta*. Our data reveal that mCherry is the RFP of choice for investigation in *Z. tritici*, showing highest signal intensity in epi-fluorescence, when used with a Cy3 filter set, and laser scanning confocal microscopy. However, mCherry bleached significantly faster than mRFP, which favors this red tag in long-term observation experiments. Finally, we used dual-color imaging of eGFP and mCherry expressing wild-type strains *in planta* and show that pycnidia are formed by single strains. This demonstrates the strength of this method in tracking the course of *Z. tritici* infection in wheat.

## Highlights

• We investigate brightness and photo-stability of RFPs in live *Z. tritici* cells.
• mCherry is most useful in epi-fluorescence and confocal laser scanning microscopy.
• The combination of mCherry and an orange-shifted filter set proves to provide brightest signals.
• We provide 4 vectors with various mRFPs for yeast recombination based cloning.
• The vectors carry carboxin resistance and integrate as single copies into the *sdi1* locus.

## 1 Introduction

The identification of red-fluorescent proteins (RFPs), with red-shifted wavelength spectra, opened a new dimension in live cell imaging. Here, the simultaneous visualization of green-fluorescent protein (GFP) and RFP allows us to track two different organelles or proteins in the same living cell (Su et al., 2004), or in different populations of particular species in a defined environment (Bloemberg et al., 2000). The first RFP described was derived from a red colored *Discosoma* coral species (Matz et al., 1999). While its excitation and emission maxima, at 558 nm and 583 nm, made it suitable for dual color imaging with GFP, DsRed oligomerizes in living cells (Baird et al., 2000). This feature limits its use as a genetic fusion tag to study protein dynamics. This caveat was overcome by genetic modification which led to a monomeric red fluorescent protein, named mRFP (Campbell et al., 2002). Further improvement of mRFP resulted in tdTomato and mCherry, which showed slightly different excitation and emission maxima, increased brightness and photo-stability (Shaner et al., 2004). The repertoire of these molecular tools was recently extended with a novel red fluorescent protein, TagRFP, from the sea anemone *Entacmaea quadricolor*, which is reported to be 3-times brighter than mCherry (Merzlyak et al., 2007).

All red fluorescent proteins described are established molecular tools with which fungi can be studied. For example, mCherry allowed expression studies in *Magnaporthe oryzae* (Saitoh et al., 2014), localization of metabolic and other enzymes in *Fusarium fujikuroi* (Albermann et al., 2013) and *Candida guilliermondii* (Courdavault et al., 2011) and effector protein secretion in host pathogen interaction in *Ustilago maydis* (Bielska et al., 2014; Djamei et al., 2011; Doehlemann et al., 2009). mRFP was used to investigate microtubule dynamics in *U. maydis* (Straube et al., 2006) and effector secretion in *M. oryzae* (Ribot et al., 2013). TagRFP revealed dynamics of the actin cytoskeleton in *Neurospora crassa* (Berepiki et al., 2010), while tdTomato visualized the grass pathogen *Ophiosphaerella herpotricha* in infected plant tissue (Caasi et al., 2010) and organelles and effector secretion in *M. oryzae* (Khang et al., 2010) and in the oomycete *Phytophthora infestans* (Kelley et al., 2010).

In this study, we aim to establish the optimal red fluorescent tag in the wheat pathogen *Zymoseptoria tritici*. We take into account that the type of microscope, illumination settings and filter sets to capture the fluorescence intensities impacts significantly on the signal brightness and rate of photo bleaching (Shaner et al., 2005). We constructed vectors for targeted single integration of vectors, carrying mRFP, TagRFP, mCherry and tdTomato, under the control of the *Z. tritici* α-tubulin promoter, placed into the defined *sdi1* locus. This allowed comparative and quantitative analysis of fluorescent brightness and photo-bleaching behavior in all RFP-expressing *Z. tritici* strains, using epi-fluorescent and confocal laser scanning microscopy. Our results demonstrate that mCherry is the optimal red fluorescent protein for studies in *Z. tritici*.

## 2 Materials and methods

### 2.1 Bacterial and fungal strains and growth conditions

*Escherichia coli* strain DH5α was used for the maintenance of plasmids. *Agrobacterium tumefaciens* strain EHA105 (Hood et al., 1993) was used for maintenance of plasmids and subsequently for *A. tumefaciens*-mediated transformation of *Z. tritici*. *E. coli* and *A. tumefaciens* were grown in DYT media (tryptone, 16 g/l; yeast extract, 10 g/l; NaCl, 5 g/l; with 20 g/l agar added for preparing the plates) at 37 °C and 28 °C respectively. The fully sequenced *Z. tritici* wild-type isolate IPO323 (Goodwin et al., 2011; Kema and van Silfhout, 1997) and another wild-type isolate IPO94269 (Kema et al., 2000) were used as recipient strains for the genetic transformation experiments. Cells were maintained as glycerol stocks (NSY glycerol; nutrient broth, 8 g/l; yeast extract, 1 g/l; sucrose, 5 g/l; glycerol, 700 ml/l), and cultures were grown on YPD agar (yeast extract, 10 g/l; peptone, 20 g/l; glucose, 20 g/l; agar, 20 g/l) at 18 °C for 4–5 days.

### 2.2 Molecular cloning

All vectors in this study were generated by *in vivo* recombination in the yeast *Saccharomyces cerevisiae* DS94 (MATα, *ura3-52*, *trp1-1*, *leu2-3*, *his3-111*, and *lys2-801* (Tang et al., 1996) following published procedures (Raymond et al., 1999; Kilaru and Steinberg, 2015). For all the recombination events, the fragments were amplified with 30 bp homologous sequences to the upstream and downstream of the fragments to be cloned (see Table 1 for primer details). PCR reactions and other molecular techniques followed standard protocols (Sambrook and Russell, 2001). The DNA fragments of interest were excised from the agarose gel and purified by using silica glass suspension as described previously (Boyle and Lew, 1995). Plasmid DNA was isolated from the positive yeast colonies as described previously (Hoffman and Winston, 1987). All restriction enzymes and reagents were obtained from New England Biolabs Inc. (NEB, Herts, UK).

### 2.3 Construction of vectors pCmRFP, pCTagRFP, pCmCherry, and pCtdTomato

The vector pCmRFP contains *mrfp* under the control of *Z. tritici tub2* promoter for integration into the *sdi1* locus by using carboxin as a selectable marker. A 12,704 bp fragment of pCeGFPTub2 (digested with *Zra*I; Schuster et al., 2015), 1149 bp *tub2* promoter (amplified with SK-Sep-14 and SK-Sep-15; Table 1) and 690 bp *mrfp* (amplified with SK-Sep-85 and SK-Sep-86; Table 1) were recombined in *S. cerevisiae* to obtain the vector pCmRFP. The vector pCTagRFP contains *tagrfp* under the control of *Z. tritici tub2* promoter for integration into the *sdi1* locus by using carboxin as a selectable marker. A 12,704 bp fragment of pCeGFPTub2 (digested with *Zra*I), 1149 bp *tub2* promoter (amplified with SK-Sep-14 and SK-Sep-15; Table 1) and 714 bp *tagrfp* (amplified with SK-Sep-81 and SK-Sep-82; Table 1) were recombined in *S. cerevisiae* to obtain the vector pCTagRFP. The vector pCtdTomato contains *tdtomato* under the control of *Z. tritici tub2* promoter for integration into the *sdi1* locus by using carboxin as a selectable marker. A 12,704 bp fragment of pCeGFPTub2 (digested with *Zra*I), 1149 bp *tub2* promoter (amplified with SK-Sep-14 and SK-Sep-15; Table 1) and 1431 bp *tdtomato* (amplified with SK-Sep-89 and SK-Sep-90; Table 1) were recombined in *S. cerevisiae* to obtain the vector pCtdTomato. The vector pCmCherry contains *mCherry* under the control of *Z. tritici tub2* promoter for integration into the *sdi1* locus by using carboxin as a selectable marker. A 12,704 bp fragment of pCeGFPTub2 (digested with *Zra*I), 1149 bp *tub2* promoter (amplified with SK-Sep-14 and SK-Sep-15; Table 1) and 714 bp *mCherry* (amplified with SK-Sep-83 and SK-Sep-84; Table 1) were recombined in *S. cerevisiae* to obtain the vector pCmCherry. The vector pHeGFP contains *egfp* under the control of *Z. tritici tub2* promoter for ectopic random integration by using hygromycin as a selection agent. A 13,534 bp fragment of pCeGFPTub2 (digested with *Bam*HI and *Bgl*II), and 1523 bp hygromycin resistance cassette (amplified with SK-Sep-128 and SK-Sep-129; Table 1) were recombined in yeast *S. cerevisiae* to obtain the vector pHeGFP. Further details on vector construction and yeast recombination-based cloning is provided in Kilaru and Steinberg (2015).

### *2.4* *Z. tritici* transformation and molecular analysis of transformants

The vectors pCmRFP, pCTagRFP, pCmCherry, and pCtdTomato were transformed into *A. tumefaciens* strain EHA105 by heat shock method (Holsters et al., 1978) and *A. tumefaciens*-mediated transformation *of Z. tritici* was performed as described previously by Zwiers and De Waard (2001) with the slight modifications. Further details on this method are provided in Kilaru et al. (2015a). To confirm the integration of vector into the *sdi1* locus and also to determine the copy number, Southern blot hybridizations were performed by using standard procedures (Sambrook and Russell, 2001). Approximately 3 μg of genomic DNA of IPO323 and transformants obtained with vectors pCmRFP, pCTagRFP, pCmCherry, and pCtdTomato were digested with *Bgl*II and separated on a 1.0% agarose gel and capillary transferred to a Hybond-N membrane (GE healthcare, Little Chalfont, United Kingdom). 1014 bp *sdi1* probe (3′ end of the *sdi1* gene and *sdi1* terminator) was generated by using DIG labelling PCR mix (Life Science Technologies, Paisley, UK) with primers SK-Sep-10 and SK-Sep-13 (Table 1). Hybridizations were performed at 62 °C for overnight autoradiographs were developed after an appropriate time period.

### 2.5 Fungal plant infection

Attached wheat leaf infections were performed, as described previously (Rudd et al., 2008) with slight modifications. Wheat cultivar Galaxie (Fenaco, Bern, Switzerland) was used for all the plant infections and further details are provided in Kilaru et al. (2015a).

### 2.6 Epi-fluorescence microscopy

Fluorescence microscopy was performed as previously described (Kilaru et al., 2015b). Different filter sets (Cy3 ET filter set, 545/25 ET Bandpass, Beam splitter T565 LPXR, 605/70 ET Bandpass; Orange Bandpass H Filterset, 544/23 H Bandpass, Laser beam splitter H560 LPXR superflat, 595/50 H Bandpass; DsRed ET Filterset, 545/30 ET Bandpass, Beam splitter T 570 LP, 620/60 ET Bandpass; mCherry HC filter set, 562/40 BrightLine HC, Laser beam splitter zt 561 RDC, 624/40 BrightLine HC: AHF Analyzentechnik AG, Tübingen, Germany) were used to analyze the average intensity and bleaching behavior of the different red tags. They were excited using a standard mercury burner and imaged in the stream acquisition mode at identical conditions. Average intensity and bleaching behavior were analyzed in the resulting movies containing 200 planes captured with 150 ms exposure time using a CoolSNAP HQ2 camera (Photometrics/Roper Scientific, Tucson, USA).

### 2.7 Confocal microscopy of liquid cultures and infected plant tissue

RFPs in cells, grown in liquid culture, were imaged using a Leica SP8 laser scanning confocal microscope (Leica, Wetzlar, Germany) equipped with a HC PL APO CS2 63×/1.40 OIL objective (Leica, Wetzlar, Germany) at 100% of the 561 nm laser (tdTomato, TagRFP) or 594 nm Laser (mRFP, mCherry). Signals were detected using HyD detectors in counting mode, which covered the emission maximum ± 10 nm (tdTomato 581 ± 10 nm, TagRFP 584 ± 10 nm, mRFP607 ± 10 nm, mCherry 610 ± 10 nm). For liquid cultures, image series of 200 planes were acquired in the stream acquisition mode, using a scan field of 256 × 256 pixels, a scan speed of 600 Hz, a zoom of 1.28 and a resolution of 16 bit. The average intensity and bleaching behavior was analyzed using the Leica LAS AF software (more details in Section 2.8).

To visualize the fungus inside plant tissue, leaf samples were collected 14 dpi, briefly dipped into Flutec PP11 (F2 Chemicals Ltd., Lea Town, UK) and placed on Carolina observation Gel (Carolina Biological Supply Company, Burlington, USA). The invasion site was identified by bright-field microscopy. Imaging was done using a Leica SP8 laser scanning confocal microscope (Leica, Wetzlar, Germany) equipped with a HC PL APO CS2 63×/1.40 oil objective (Leica, Wetzlar, Germany). The average intensity and bleaching behavior *in planta* was investigated at 100% output power of the 561 nm laser (tdTomato, TagRFP) or 594 nm Laser (mRFP, mCherry), using identical HyD detector settings as described above. Image series of 150 planes were acquired, using a scan field of 512 × 512 pixels, a scan speed of 600 Hz, a zoom of 2 and bit depth of 12 bit, using the stream acquisition mode. To acquire images for the Fig. 3c of this publication, mCherry and TagRFP expressing fungi were visualized using a HC PL APO CS2 40×/1.30 oil objective (Leica, Wetzlar, Germany). Samples were exited using 100% of the 561 nm laser (TagRFP) or 594 nm Laser (mCherry) and signals were taken in the standard mode of the HyD detectors, opened at 584 ± 10 nm (TagRFP), 610 ± 10 nm (mCherry), with a gain of 300, a scan field of 1024 × 1024 pixels, a scan speed of 400 Hz, a zoom of 1, a line average of 2 and a image bit depth of 12 bit. Auto-fluorescence of the chloroplasts and cell walls was detected using a second HyD detector at a gain of 28.

Co-visualization of fungi expressing mCherry and eGFP was done using a Leica SP8 laser scanning confocal microscope (Leica, Wetzlar, Germany), equipped with a HC PL APO CS2 40×/1.30 oil objective (Leica, Wetzlar, Germany), using an argon laser at 10% and the 594 nm laser at 20% output power. GFP and mCherry fluorescence was detected using HyD detectors in standard mode at a gain of 161 and 199, respectively. A scan field of 1024 × 1024 pixels, a scan speed of 400 Hz, a zoom of 1, a line average of 2 and a image bit depth of 8 bit was used to capture z-Stacks over 10 μm depth with a *z*-resolution of 0.5 μm.

### 2.8 Data analysis

The average intensity of the different red tags in the fungal cytoplasm was analyzed by creating one region of interest (ROI) per cell, in the first plane of the generated streams, covering only a part of the cytoplasm but excluding the nucleus or vacuoles. A copy of the same ROI was placed next to the cell to acquire the average intensity of the neighboring background. The values of both ROI’s from the first plane of the movie were transferred to Excel (Microsoft, Redmond, WA, USA) and the values of the neighboring background ROI’s were subtracted from the ROI’s of the fungal cytoplasm. All corrected values were transferred to Prism 5.03 (GraphPad Software, La Jolla, CA, USA) to perform intensity comparisons. All statistical testing was done using Prism 5.03 (GraphPad Software, La Jolla, CA, USA).

In order to analyze the bleaching behavior, bleaching curves were generated. To this end, the average intensity of the different red tags in the fungal cytoplasm was measured in one region of interest (ROI) per cell, covering an area of cytoplasm, but excluding organelles, such as the nucleus or vacuoles. The area of the ROI was moved out of the cells to acquire the average intensity of the neighboring background. The measured intensity values of both ROI’s from each plane of the movie were transferred to Excel (Microsoft, Redmond, WA, USA), and the average intensity value of the extracellular background was subtracted from the average signal intensity in the cellular ROI. This was done for numerous cells and the mean ± standard deviation of the corrected intensities for each plane was calculated. Curves were drawn in the program Prism 5.03 (GraphPad Software, La Jolla, CA, USA).

To compare the rate by which bleaching occurs in the various RFPs, the measured fluorescent intensity values with time were fitted to a one phase decay model. Decay curves were compared using *F* testing the best fitting decay rates between individual data sets. Fitting and *F* testing are performed in the software Prism 5.03 (GraphPad Software, La Jolla, CA, USA).

## 3 Results and discussion

### 3.1 Vectors for targeted ectopic integration of RFP-encoding constructs

**Fig. 1:**
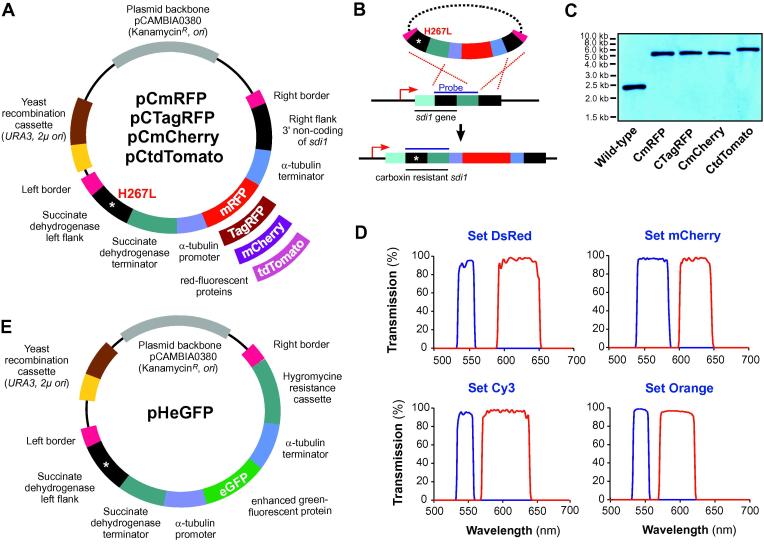
Vectors for integration of various red-fluorescent proteins into the genome of *Z. tritici*. (A) Cloning vectors for controlled integration of various RFPs into the *sdi1* locus of *Z. tritici*. TagRFP: generated from the wild-type RFP from sea anemone *E. quadricolor* (Merzlyak et al., 2007); mRFP: a derivative of the red fluorescent protein from *Discosoma* corals (Campbell et al., 2002); tdTomato and mCherry: mutated versions of the red fluorescent protein from *Discosoma* corals (Shaner et al., 2004). After integration into the *sdi1* locus, the vector confers carboxin resistance due to a point mutation in the succinate dehydrogenase gene *sdi1*, which changes a histidine to a leucine (H267L). For more details of this integration into the “carboxin locus” (Kilaru et al., 2015a). Left and right border enable *Agrobacterium tumefaciens*-based transformation of *Z. tritici*. Note that fragments are not drawn to scale. For more accurate information on fragment sizes see main text. (B) Image illustrates the integration of any vector shown (A) into the native *sdi1* locus of *Z. tritici*. This co-integrates a carboxin-resistant *sdi1*^H267L^ allele and cytoplasmic RFPs, expressed under the control of α-tubulin promoter (*tub2*). (C) Southern blot, showing integration of vectors into the *sdi1* locus. After digestion of the genomic DNA with *Bgl*II and subsequent hybridisation with a labelled DNA probe, a shift in the DNA fragments from 2.3 kb to 5.3 kb and 6.0 kb is detected. Size standards are given at the left. (D) Transmission spectra of the emission and excitation filters of various filter sets, tested in this study. For details see main text. (E) Cloning vector for ectopic integration of enhanced GFP for cytoplasmic expression in *Z. tritici*. The vector is compatible with yeast recombination-based cloning, expresses cytoplasmic eGFP and confers resistance to hygromycin. Note that the vector pHeGFP was derived from carboxin resistance conferring vector pCeGFP (Kilaru et al., 2015a, 2015c). As such they contain part of the succinate dehydrogenase gene, carrying the mutation H267L and succinate dehydrogenase terminator. However, these fragments are of no significance.

As a first step toward finding the optimal red florescent protein for use in *Z. tritici*, we generated 4 vectors, pCmRFP, pCTagRFP, pCmCherry, pCtdTomato, which express mRFP, TagRFP, mCherry and tdTomato in the cytoplasm under the control of the constitutive *Z. tritici* α-tubulin (*tub2*) promoter (Fig. 1A; for details on *tub2* see Schuster et al., 2015). These vectors were designed for targeted integration into the genomic *sdi1* locus of *Z. tritici*, by using a mutated downstream stretch of the *sdi1* sequence, carrying a carboxin resistance conferring point mutation (H267L; Fig. 1A, left flank), and a sequence stretch downstream of *sdi1* (Fig. 1B, right flank of *sdi1*). Incorporation by homologous recombination mutates the *sdi1* gene and integrates the RFP constructs into the *sdi1* locus (Fig. 1B; for details see Kilaru et al., 2015a). This results in comparable gene expression due to an identical genomic environment and single integration of each construct, which is essential for quantitative analysis of fluorescent intensities. All four vectors were built on the *Agrobacterium* binary vector pCAMBIA0380 (CAMBIA, Canberra, Australia), which allows *A. tumefaciens*-based transformation into *Z. tritici*, based on the 25 bp imperfect directional repeat sequences of the T-DNA borders (right and left border, RB and LB; Fig. 1A). The vector also carries a kanamycin resistance gene, origin of replications for *E. coli and A. tumefaciens* and a “yeast recombination cassette”, consisting of URA3 and 2μ *ori* which enables yeast recombination-based cloning (for more details see Kilaru and Steinberg, 2015).

We next transformed all four vectors into *Z. tritici* strain IPO323 (Kema and van Silfhout, 1997) using *A. tumefaciens*-mediated transformation (Zwiers and De Waard, 2001). In order to confirm the single copy integration into the *sdi1* locus, we purified genomic DNA from the transformants and wild-type isolate IPO323, digested with *Bgl*II and hybridised to a *sdi1* probe. In all cases, we found a single band at the expected sizes (5.3 kb for pCmRFP, pCTagRFP, pCmCherry and 6.0 kb for pCtdTomato; Fig. 1C), confirming that all RFP constructs were integrated into the *sdi1* locus as single copies. This resulted in strains IPO323_CmRFP, IPO323_CTagRFP, IPO323_CmCherry and IPO323_CtdTomato respectively. None of these RFP-expressing strains was affected in virulence (see Kilaru et al., 2015a), demonstrating that cytoplasmic expression of mRFP, TagRFP, mCherry or tdTomato is not toxic to the cells.

The correct choice of filter sets impacts significantly on the brightness and rate of photo-bleaching of fluorescent proteins (Shaner et al., 2005). While mRFP, mCherry and tdTomato are derived from the same *Discosoma* protein (Matz et al., 1999; Shaner et al., 2004), the introduced mutations altered their emission and excitation spectra (maximum excitation/emission: DsRed 558 nm/583 nm, mRFP 584 nm/607 nm, mCherry 587 nm/610 nm, tdTomato 554 nm/581 nm). Similar to tdTomato, *E. quadricolor* TagRFP has its maximum excitation at 555 nm and the maximum emission at 584 nm (Merzlyak et al., 2007). Consequently, we decided to test all RFPs in combination with various filter sets to find the optimal combination for studies in *Z. tritici*. We received four filter sets with slightly different optical spectra (Fig. 1D; Cy3 ET filter set = Set-Cy3, Orange Bandpass H Filterset = Set-Organe, DsRed ET filter set = Set Ds-Red; mCherry HC filter set = Set-mCherry; provided by AHF Analyzentechnik AG, Tübingen, Germany; for technical details see Section 2).

### 3.2 Fluorescent behavior of mRFP, TagFRP, mCherry and tdTomato in epi-fluorescence microscopy

**Fig. 2:**
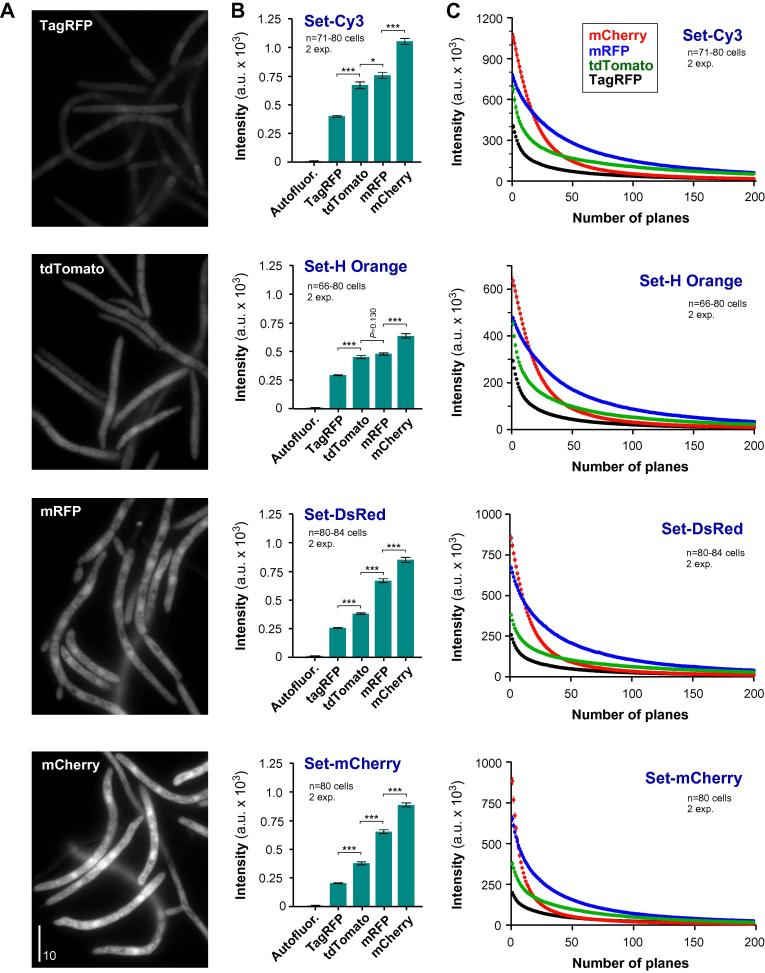
Signal intensity and bleaching behavior of red-fluorescent proteins in epi-fluorescence microscopy. (A) Images showing cytoplasmic expression of TagRFP, mRFP, tdTomato and mCherry. Note that *Z. tritici* shows virtually no auto-fluorescence (see B). All images were acquired and processed identically. Bar represents 10 μm. (B) Bar chart showing intensity of cytoplasmic fluorescence of various RFPs in 4 different filter sets (see Fig. 1E for fluorescent spectra). Autofluor.: background fluorescence without expressing a RFP; TagRFP: a mutant protein, generated from the wild-type RFP from sea anemone *E. quadricolor*; mRFP: a derivative of the red fluorescent protein from *Discosoma* corals; tdTomato and mCherry: mutated versions of the red fluorescent protein from *Discosoma* corals. Mean ± standard error of the mean is shown, sample size *n* (=number of cells) is indicated. Single asterisk indicates significant difference at *P* = 0.0282, triple asterisk at *P* < 0.0001, Student *t*-test. (C) Graph showing decay of fluorescent signals due to photo-bleaching in 4 fluorescent filter sets. TagRFP: a mutant protein, generated from the wild-type RFP from sea anemone *E. quadricolor*; mRFP: a derivative of the red fluorescent protein from *Discosoma* corals; tdTomato and mCherry: mutated versions of the red fluorescent protein from *Discosoma* corals. Each data point is given as mean ± standard error of the mean, sample size *n* (=number of cells) is indicated. Note that mRFP is most stable whereas mCherry shows the brightest signal.

Having generated four *Z. tritici* strains, expressing non-toxic red fluorescent proteins from the same locus and under the same *tub2* promoter, enabled a quantitative comparison of their fluorescent brightness and photo-bleaching behavior. In a first set of experiments, we tested these two variables in epi-fluorescence microscopy, using four orange/red florescent filter sets. We investigated one day old liquid cultures of IPO323 control cells and red fluorescent protein expressing strains (IPO323_CmRFP; IPO323_CTagRFP; IPO323_CmCherry; IPO323_CtdTomato) using a HBO mercury short-arc lamp for excitation and identical acquisition settings. Under these conditions, all RFP-expressing strains showed good levels of cytoplasmic and dim nuclear fluorescence (Fig. 2A; images shown were taken using mCherry HC filter set). Untransformed IPO323 cells showed no significant auto-fluorescence (Fig. 2B). From the quantitative image analysis, we found that mCherry provided the brightest signal in all filter sets (Fig. 2B). Interestingly, maximum signal intensity was found when mCherry was combined with the Cy3 ET filter set. This is surprising, considering the filter spectra and the emission and excitation curves of mCherry. The mCherry HC filter set is adapted to cover most of the range of light excitation of mCherry (∼70% better than the Cy3 ET filters) and collects ∼20% more of the emission light. However, it is important to realize that the properties of fluorescent proteins depend on the environment. Emission and excitation spectra measured *in vitro* differ from those in living cells (Wack et al., 2003). In fact, the brightness of mCherry was shown to vary in the same cell, depending on the cell cycle stage (Doherty et al., 2010). This could be due to pH variations or various folding stages of a fluorescent protein (Doherty et al., 2010; Hebisch et al., 2013; Pineda Rodo et al., 2012). While the underlying reason is unclear, our data show that the combination of mCherry and the Cy3 ET filter provides the highest signal intensity, when used in *Z. tritici*.

We next investigated photo-bleaching behavior of all RFPs with all four filter sets. We continuously illuminated the cells using HBO illumination and stream acquisition and measured the decay of fluorescent signal intensity with time. Fluorescence of all RFPs decreased with time, indicating that all fluorescent proteins undergo photo-bleaching (Fig. 2C; exposure time per plane: 150 ms). Using different filter sets, we found that mCherry was bleaching fast and, in all cases, performed worse than tdTomato and mRFP (significantly more rapid decay than tdTomato and mRFP in all filters; *P* < 0.0001 for *F* testing of curves after fitting to one way decay). Most rapid signal decay was found when mCherry was observed using the mCherry HC filter set (decay coefficient *α* = 0.094 ± 0.0004), whereas it was more photo-stable when the Cy3 ET filter set was used (decay coefficient *α* = 0.04725 ± 0.00022). In all filters used, mRFP fluorescence decayed relatively slow (Fig. 2C; all decay coefficients *α* range from 0.032 ± 0.0002 to 0.02249 ± 0.00022), suggesting that this red tag is suitable for long-term observations. In summary, we conclude that mCherry is best-suited for short-term observation of fluorescent signals. However, mRFP fluorescence is ∼70% as bright as that of mCherry, but signals bleach less quickly. Therefore, mRFP may serve as an alternative red tag, with advantages in long term observation experiments. Alternatively, one may consider using mCherry at reduced excitation light. While this would reduce the signal intensity, it may improve the bleaching behavior and make up for this disadvantage.

### 3.3 Fluorescent behavior of mRFP, TagFRP, mCherry and tdTomato in confocal laser scanning microscopy

**Fig. 3:**
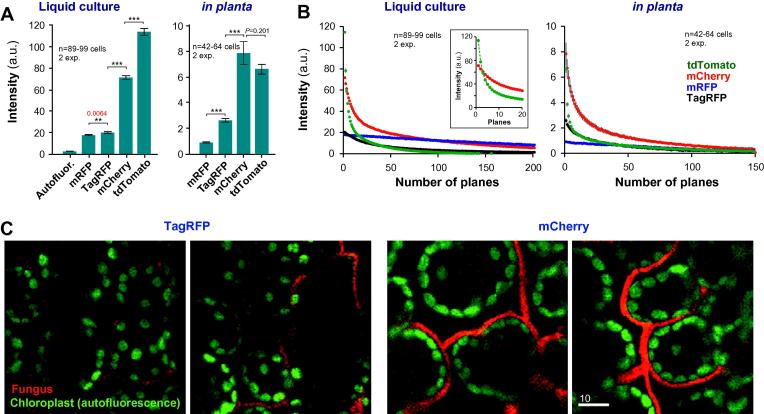
Signal intensity and bleaching behavior of RFP proteins in confocal laser-scanning microscopy. (A) Bar charts showing intensity of cytoplasmic fluorescence of various RFPs, observed with a confocal laser scanning microscope in liquid culture and in infected wheat tissue (*in planta*). Autofluor. = background fluorescence without expressing a RFP; TagRFP = a mutant protein, generated from the wild-type RFP from sea anemone *E. quadricolor*; mRFP = a derivative of the red fluorescent protein from *Discosoma* corals; tdTomato and mCherry = mutated versions of the red fluorescent protein from *Discosoma* corals. Mean ± standard error of the mean is shown, sample size *n* (=number of cells) is indicated. Double asterisk indicates significant difference at *P* = 0.0064, triple asterisk indicates significant difference at *P* < 0.0001, Student *t*-test. (B) Graph showing decay of fluorescent signals due to photo-bleaching in confocal laser scanning microscopy, both in infected wheat tissue (*in planta*) and in liquid culture (Liquid culture). TagRFP: a mutant protein, generated from the wild-type RFP from sea anemone *E. quadricolor*; mRFP: a derivative of the red fluorescent protein from *Discosoma* corals; tdTomato and mCherry: mutated versions of the red fluorescent protein from *Discosoma* corals. Each data point is given as mean ± standard error of the mean, sample size *n* (=number of cells) is indicated. In confocal microscopy, tdTomato shows the brightest signal, but undergoes rapid decay due to photo-bleaching (see inset). (C) Images of infected wheat tissue at 14 dpi. Hyphal cells express cytoplasmic TagRFP and mCherry. Bar represents 10 μm.

Confocal laser scanning microscopy has proven to be a powerful method to investigate host pathogen interactions (e.g. Djamei et al., 2011; Doehlemann et al., 2009; Macia-Vicente et al., 2009). We therefore tested the use of RFPs in liquid culture and *in planta* using a Leica TCS SP8L confocal microscope. We found that mRFP and TagRFP gave very faint signals, both in fungal cells in liquid culture and in infected plant tissue (Fig. 3A and C), while mCherry provided strong fluorescence and mCherry expressing hyphae were clearly visible *in planta* (Fig. 3A and C). Thus, mRFP and TagRFP are not suitable for investigating *Z. tritici* in plant infection studies. In contrast to the results for epi-fluorescence, tdTomato showed the brightest fluorescent intensity in liquid culture (Fig. 3A) and was almost as bright as mCherry *in planta*. However, tdTomato was rapidly photobleached when observed in laser scanning microscopy (Fig. 3B, decay coefficient *α*_liquid_ = 0.1524 ± 0.00118; decay coefficient *α_in planta_* = 0.1614 ± 0.0033), whereas mCherry fluorescence decayed ∼3–5-times slower (Fig. 3B; decay coefficient *α*_liquid_ = 0.03502 ± 0.00031; decay coefficient *α_in planta_* = 0.0576 ± 0.0015). Taken together, these results show that mCherry is best suited for investigation of *Z. tritici* in epi-fluorescent and confocal laser scanning microscopy.

### 3.4 Dual-color microscopy of *Z. tritici* in infected wheat leaves

Establishing mCherry as the optimal red-fluorescent proteins allowed dual color imaging with green-fluorescent proteins, which could be useful to understand the infection biology of *Z. tritici*. Hilu and Bever (1957) described that pycnidia are formed after invasion of the stomatal cavity by a single hypha. We aimed to test this by labelling two different wildtype strains with eGFP and mCherry. If pycnidia are formed by a single hypha, we expected to find either red or green fluorescent fungal material beneath a stomata. However, if both participate in pycnidium formation, we should see both colors represented in the mycelium. To this end, we generated vector pHeGFP (Fig. 1E), which carries a hygromycin B resistance cassette, is compatible with yeast recombination-based cloning and allows cytoplasmic enhanced GFP (Yang et al., 1996) expression under the *tub2* promoter, after random integration into the genome of *Z. tritici*. Note that the vector pHeGFP was derived from carboxin resistance conferring vector pCeGFP (Kilaru et al., 2015a and 2015c). As such, they contain part of the succinate dehydrogenase gene, carrying the mutation H267L and succinate dehydrogenase terminator. However, these fragments are of no significance.

**Fig. 4:**
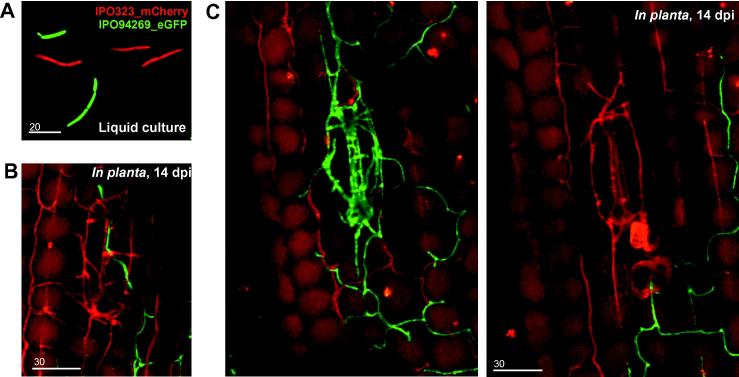
Co-visualization of *Z. tritici* strain IPO323, expressing mCherry and IPO94269, expressing eGFP. (A) Image showing mCherry and eGFP fluorescence of both strains in liquid culture. Bar represents 20 μm. (B and C) Images showing mCherry and eGFP fluorescence of both strains in infected wheat tissue at 14 dpi. Note that hyphae of either strain colonize the stomatal cavity to form pre-pycnidia. Either IPO323 or IPO94269 was found (B), suggesting that they mutually exclude each other. Occasionally, single hyphae were crossing a colonized stomata space (C). Bar represents 30 μm.

Integration of the vector pHeGFP into the wild-type strain IPO94269 (Kema et al., 2000) resulting in strain IPO94269_HeGFP. After co-infection of wheat plants with a mixture of IPO94269_HeGFP and IPO323_CmCherry, individual cells of both strains were identified by the expression of the red and green fluorescent proteins (Fig. 4A). At 14 days post infection, we found mCherry- and eGFP-expressing fungal hyphae in the same field of observation (Fig. 4B). However, stomatal cavities were colonized by either IPO323_CmCherry (Fig. 4C; red) or by IPO94269_HeGFP (Fig. 4C; green). Occasionally, both strains appear at a sub-stomatal opening, but no mixed mycelium was found. These results are consistent with the findings that single hypha forms a pycnidium (Hilu and Bever, 1957). This illustrates the potential of having established the ability for dual-color microscopy in *Z. tritici*.

## 4 Conclusion

In this study we established genetically encoded red fluorescent tags for *Z. tritici*. Extensive quantitative analysis of fluorescent brightness and photo-bleaching behavior, both in liquid culture and in infected plant tissue, shows that mCherry is the optimal RFP for use in *Z. tritici*. However, mRFP shows advantages in long-term observation of using epi-fluorescence microscopy, as it is more photostable. tdTomato provided brighter signals in confocal microscopy, but bleached rapidly. Having established a red fluorescent tag, dual-color imaging using GFP is possible. As part of this study, we visualized two FP tagged wild-type strains in co-infected wheat leaves. This demonstrates that stomata openings are colonized predominantly by a single strain. This example illustrates the potential strength of the methods for understanding principles of wheat infection by *Z. tritici*.

## Figures and Tables

**Table 1:** Primers used in this study.

Primer name	Direction	Sequence (5′–3′)^a^
SK-Sep-10	Sense	*TGGCAGGATATATTGTGGTGTAAACAAATT*GACCTTCCACATCTACCGATGG
SK-Sep-13	Antisense	CTTCCGTCGATTTCGAGACAGC
SK-Sep-14	Sense	*CATTTGCGGCTGTCTCGAAATCGACGGAAG*GCAGTCGACGCCAGATGATGG
SK-Sep-15	Antisense	*GGTGAACAGCTCCTCGCCCTTGCTCACCAT*GGCGATGGTGGTATGCGGATG
SK-Sep-81	Sense	*CATCACTCACATCCGCATACCACCATCGCC*ATGGTGTCTAAGGGCGAAGAGC
SK-Sep-82	Antisense	*CCACAAGATCCTGTCCTCGTCCGTCGTCGC*CTAATTAAGTTTGTGCCCCAGTTTGCTA
SK-Sep-83	Sense	*CATCACTCACATCCGCATACCACCATCGCC*ATGGTGAGCAAGGGCGAGGAGG
SK-Sep-84	Antisense	*CCACAAGATCCTGTCCTCGTCCGTCGTCGC*TTACTTGTACAGCTCGTCCATGCC
SK-Sep-85	Sense	*CATCACTCACATCCGCATACCACCATCGCC*ATGGGCCGTTCCTCCGAGGAC
SK-Sep-86	Antisense	*CCACAAGATCCTGTCCTCGTCCGTCGTCGC*TTACTTGTACAGGGCGCCGGTG
SK-Sep-89	Sense	*CATCACTCACATCCGCATACCACCATCGCC*ATGGTGAGCAAGGGCGAGGAGGT
SK-Sep-90	Antisense	*CCACAAGATCCTGTCCTCGTCCGTCGTCGC*TTACTTGTACAGCTCGTCCATGCCG
SK-Sep-128	Sense	*CTCTCATAAGAGCTTGGCTGTCGACTCCTC*GAATTCGAGCTCGGTACCCAACT
SK-Sep-129	Antisense	*CTTTTCTCTTAGGTTTACCCGCGTTGAAGT*GCGTTAACACTAGTCAGATCTACC

a *Italics* indicate part of the primer that is complementary with another DNA fragment, to be ligated by homologous recombination in *S. cerevisiae*.
